# Mechanisms of Action Involved in Ozone Therapy: Is healing induced via a mild oxidative stress?

**DOI:** 10.1186/2045-9912-1-29

**Published:** 2011-12-20

**Authors:** Masaru Sagai, Velio Bocci

**Affiliations:** 1 Tsukuba Institute for Healthy-Life, Higashi Hiratuka 586-2, Tsukuba, Ibaraki, Japan; 2 Department of Physiology, Viale A. Moro 2, 53100, University of Siena, Italy

**Keywords:** ozone therapy, oxidative stress, NFκB activity, Nrf2 activity, antioxidant response element (ARE), nuclear factor of activated T cells (NFAT), activated protein-1 (AP-1), hypoxia inducible factor-1α (HIF-1α)

## Abstract

The potential mechanisms of action of ozone therapy are reviewed in this paper. The therapeutic efficacy of ozone therapy may be partly due the controlled and moderate oxidative stress produced by the reactions of ozone with several biological components. The line between effectiveness and toxicity of ozone may be dependent on the strength of the oxidative stress. As with exercise, it is well known that moderate exercise is good for health, whereas excessive exercise is not.

Severe oxidative stress activates nuclear transcriptional factor kappa B (NFκB), resulting in an inflammatory response and tissue injury via the production of COX2, PGE2, and cytokines. However, moderate oxidative stress activates another nuclear transcriptional factor, nuclear factor-erythroid 2-related factor 2 (Nrf2). Nrf2 then induces the transcription of antioxidant response elements (ARE). Transcription of ARE results in the production of numerous antioxidant enzymes, such as SOD, GPx, glutathione-s-transferase(GSTr), catalase (CAT), heme-oxygenase-1 (HO-1), NADPH-quinone-oxidoreductase (NQO-1), phase II enzymes of drug metabolism and heat shock proteins (HSP). Both free antioxidants and anti-oxidative enzymes not only protect cells from oxidation and inflammation but they may be able to reverse the chronic oxidative stress. Based on these observations, ozone therapy may also activate Nrf2 via moderate oxidative stress, and suppress NFκB and inflammatory responses. Furthermore, activation of Nrf2 results in protection against neurodegenerative diseases, such as Alzheimer's and Parkinson's diseases. Mild immune responses are induced via other nuclear transcriptional factors, such as nuclear factor of activated T-cells (NFAT) and activated protein-1 (AP-1).

Additionally, the effectiveness of ozone therapy in vascular diseases may also be explained by the activation of another nuclear transcriptional factor, hypoxia inducible factor-1α (HIF-1a), which is also induced via moderate oxidative stress. Recently these concepts have become widely accepted. The versatility of ozone in treating vascular and degenerative diseases as well as skin lesions, hernial disc and primary root carious lesions in children is emphasized. Further researches able to elucidate whether the mechanisms of action of ozone therapy involve nuclear transcription factors, such as Nrf2, NFAT, AP-1, and HIF-1α are warranted.

## Introduction

Ozone therapy, or more specifically, the major ozonated autohemotherapy (O_**3**_-AHT), has been used for almost 40 years. The first report on ozone therapy was published by Wolff in 1974 [1]. Although ozone therapy is now used all over the world, it has not yet been accepted as orthodox medicine in all countries.

About two decades ago, both nitrogen oxide (NO) and carbon monoxide (CO) were only considered as toxic air pollutants or gases derived from cigarette smoke. However, today, they are regarded to as essential gases, since both NO and CO are responsible for very important physiological actions within the body [2-4]. Similarly hydrogen sulfide, a toxic gas, is now used as a drug for the treatment of osteoporosis [5,6]. Additionally, low dose radiation was also reported to have beneficial effects within radiated cells, in particular, by prolonging the lifespan of cells via the hormesis mechanism [7]. Thus, our understanding of toxic compounds and their effects within the body appears to be constantly changing as we have realized that toxicity depends entirely on the dosage.

Both exercise and caloric restriction are excellent examples of the hormetic effect [8]. It is well known that moderate exercise is beneficial for health. Furthermore, caloric restriction is also well known to delay disease onset and mortality [9], to promote health, and to increase longevity by inducing sirtuin 1 (SIRT1), a gene responsible for longevity [10].

One of the purposes of this review is to propose a hypothesis on the mechanisms of action of ozone from the view point of oxidative stress and nuclear transcription factors, because oxidative stress acts as a second messenger in various intracellular signaling pathways. Furthermore, cells can quickly induce biological responses against oxidative stress to maintain biological homeostasis and adapt to such stresses. And, some nuclear factors induce various biological responses against oxidative stress.

The findings of animal experiments and studies on the clinical applications of ozone therapy are included. Additionally, we explore the possibility that the mechanisms of action of ozone therapy may be via the activation of antioxidant protection systems, where moderate oxidative stress may induce the activation of nuclear transcriptional factors such as nuclear factor-erythroid 2-related factor 2 (Nrf2), hypoxia inducible factor-1α(HIF-1α), nuclear factor of activated T-cells (NFAT), and activated protein-1 ( AT-1).

## 1. The ozone paradox: is ozone always toxic?

### 1.1. Reaction with ozone and biological components

Pryor et al [11] have previously described the mechanisms involved in ozone lung toxicity. Briefly, inhaled ozone reacts with polyunsaturated fatty acids (PUFA), which are found in the lipids of the alveolar lining layer (ALL), to produce ozone-specific products, referred to as lipid ozonation products (LOPs). Ozone can also react with unsaturated fatty acids to produce Criegee ozonide in the absence of H_2_O. However, in the presence of H_2_O, aldehydes and hydrogen peroxide (H_2_O_2_) are produced [11]. Since H_2_O is abundant within the pulmonary system, the main reaction with ozone will be the formation of aldehyde and H_2_O_2 _products. The net reaction is as follows:

$${\text{H}}_{\text{2}}{\text{O}}_{\text{2}}\;+\;\text{F}{\text{e}}^{++}------\to \;{\text{OH}}^{\circ }\;+\;\text{O}{\text{H}}^{-}\;+\;\text{F}{\text{e}}^{+++}$$

LOPs are proposed to be the more likely species to act as signal transduction molecules. These products may activate specific lipases, such as phospholipase A2 or phospholipase C, to release arachidonic acid (AA). In fact, AA levels increase more than 10-fold in endobronchial washings obtained from rats exposed to 1.1 ppm of ozone for 5 days [12]. The released AA can then be converted into other chemical mediators, such as various prostaglandins (PGs) and platelet activating factors (PAF), via cyclooxygenases (COXs) and lipoxygenases (LOXs) to induce an inflammatory response. Furthermore, it has been reported that 4-hydroxynonenal (4-HNE), the most toxic type of aldehyde [13,14] and H_2_O_2 _[15,16], have the ability to partake in signal transduction.

### 1.2. Effects of ozone on the airways: Airway hyperreactivity and inflammation

Airway hyper reactivity is one of the earlier signs of ozone inhalation. An increase in airway hyperreactivity is accompanied by airway inflammation. These changes are similar to those by cigarette smoking. Lin et al [17] proposed the signaling mechanisms behind airway inflammation induced via smoking. Briefly, it was demonstrated that cigarette smoke extract (CSE) added into a cell culture system of human tracheal smooth muscle cells binds to Toll-like receptor 4 (TLR4). TLR4 then activates NADPH-oxidase via adaptor molecules, such as MyD88, to produce reactive oxygen species (ROS). Further, ROS activate mitogen activated protein kinases (MAPKs), and MAPK phosphorylates IκBα part of the nuclear factor kappa B and IκBα (NFκB- IκBα) complex found in cytosol fractions. As a result NFκB is released from the complex, and this free NFκB enters into nuclei to activate the COX2 gene. COX2 then produces PGE2, which stimulates a number of cytokines, among which TNFα and IL-6. The eicosanoide and cytokines then induce the airway inflammation during the early stage of exposure. These reactions also occur in airway epithelial cells, endothelial cells, and macrophages. This signaling cascade is also supported by the findings of Williams et al. [18]. They demonstrated that TRL2 or TLR4 knockout mice do not have airway hyperreactivity and neutrophil infiltration even after an exposure to ozone of 3 ppm. These observations strongly suggest that there is a strong relationship between inflammation and TLR.

There are good experimental [19,20] and clinical [21] studies showing that exposure by inhalation to prolonged tropospheric ozone damages the respiratory system and extra pulmonary organs. The skin, if extensively exposed, may also contribute to the damage [22,23]. Consequently *the strong reactivity of ozone, *which has an electrochemical potential value, E_°_= +2.076V, *has contributed to establish the dogma that ozone is always toxic and its medical application must be proscribed. *However it will be shown that this dogma is not supported by comparing the action of ozone on the lung surface versus human blood. Obviously it must be said that ozone must be never inhaled by anyone in the clinic. However, it seems that ozone may be produced in our body similar to NO, CO and H_2_S: it has been reported that antibody-catalyzed water-oxidation pathway produced an additional molecular species with a chemical signature to that of ozone [24]. This species is also generated during the oxidative burst of activated human neutrophils and during inflammation [25].

The fundamental points to consider are: the topography, anatomical and biochemical characteristics of the organs daily exposed to ozone versus the potent antioxidant capacity of blood exposed to a small and precisely calculated dose of ozone for a few minutes. It is clear how the respiratory system undergoing a chronic oxidative stress can release slowly, but steadily, a huge amount of already mentioned toxic compounds able to act locally and to enter the circulation and cause serious damage.

### 1.3. Origin, distribution and fate of toxic compounds released by the pulmonary system during and after ozone exposure

At the airspace level, the alveolar cells are constantly overlaid by a film composed of water, salts and a myriad of biomolecules such as a profusion of surfactant phospholipids and a very small amounts of proteins, lipophilic and hydrophilic antioxidants. Any inspired gas, depending upon its relative concentration and pressure, must first dissolve into the aqueous layer before reaching the alveolar microcirculation and the erythrocytes. This process implies a physical transport regulated by a pressure gradient and a diffusion process. On the other hand, it is known that ozone, in contact with biological water, does not follow Henry's law and, although it is ten fold more soluble than oxygen, it is not transferred into the alveolar capillaries because it reacts immediately with the biomolecules present in the Alveolar Lining Layer (ALL). *It must emphasized that the average thickness of ALL is only 0.2 micron *[26]. As it was hypothesized [11], ozone does not penetrate the cells but oxidizes available antioxidants and reacts instantaneously with surfactant's polyunsaturated fatty acids (PUFA) present at the interface to form Reactive Oxygen Species (ROS), such as hydrogen peroxide and a mixture of heterogenous LOPs including lipoperoxyl radicals, hydroperoxides, malonyldialdeyde, isoprostanes, the ozonide and alkenals, particularly 4-HNE [27-29]. As cholesterol is a component of the epitherial lining fluids (ELF) and because its double bond is readily attacked by ozone, it can give rise to biologically active oxysterols [30,31] of which 3β-hydroxy-5-oxo-5,6-secocholestan-6-al (CSeco) has been implicated in pulmonary toxicity, Alzheimer's disease and atherosclerosis.

The antioxidant capacity present in the human ALL is extremely limited and, although different portions of the respiratory tract may have different antioxidant levels, these are always irrelevant in comparison to the amount of antioxidants that, in blood, easily tame the ozone reactivity. First of all, by considering the expanse of the alveolar surface (1 m^2^/kg body weight) in a 70 kg human, it can be calculated that the normal volume of ALL ranges only between 17 and 25 ml, whereas 5 L of blood include about 2.7 L of plasma. Moreover, the erythrocyte mass, amounting to about 2.3 kg, has an enormous antioxidant capacity due to hydro-lipophilic antioxidants and enzymes able to reduce any antioxidant in a few minutes [32]. Erythrocytes, via glucose-6-phosphate dehydrogenase activity in the pentose cycle, can continuously supply NADPH-reducing equivalents. The amount of plasma albumin acting as a "sacrificial compound" against oxidants is impressive (99.9% higher than in ALL). Moreover erythrocytes have a GSH content of about 2.2 mM (almost 800-fold higher than plasma) and therefore they contain a huge reserve. In the course of evolution, aerobic organisms have developed a sophisticated antioxidant system against oxygen and, although about 2% of the inhaled oxygen generates superoxide anion, this is normally neutralized at an alveolar pO_2 _pressure of 100 mm Hg. It is useful, however, to bear in mind that rats inhaling pure oxygen (alveolar pressure at about 700 mmHg) die within 60-66 h [33], Ozone is far more reactive than oxygen, and breathing air containing 10.0 ppm ozone causes death within 4 h in rats. In order to understand the effects of a daily 8-hour ozone exposure (April-October), we need to know the average environmental ozone levels that vary considerably for many reasons. The US Clean Air Act has set an ozone level of 0.06 ppm as an 8-h mean concentration to protect the health of workers (U.S. Environmental Protection Agency, 2005). Evaluation of recent studies [34,35] allows establishing an average environmental ozone concentration of 0.09 ± 0.01 ppm. However, ozone concentration in urban air can exceed 0.8 ppm in high pollution conditions [27]. For 8 h at rest (a tidal volume of about 10 l/min and a retention of inspired ozone of no less than 80%), the ozone dose amounts to 0.70-0.77 mg daily or 21.0-23.1 mg monthly. This is likely the minimal ozone intake because physical activity increases the volume of inhaled air and, at peak time, the ozone levels can easily augment to 200-300 ppb, reducing pulmonary functions and enhancing the risk of cardiovascular death [21,34-36]). Moreover, the toxicity is certainly augmented by the presence of NO_2_, CO, SO_2 _and particulate matters (PM10). On this basis, it appears clear how the ozone generated ROS and LOPs at the ALL level, after being minimally quenched by the insufficient, antioxidants will act as cell signals able to activate nuclear factor-kappa B (NF-κB), NO-synthase, some protein kinases, thus enhancing the synthesis and release of TNFα, IL-1, IL-8, IFNγ and TGFβ1 and the possible formation of nitrating species. With an increasing inflow into the alveolar space of neutrophils and activated macrophages, a vicious circle will start, perpetuating the production of an excess of ROS including also hypoclorous acid [37,38]. Moreover, during Summer, there is a continuous flow of ozone entering the respiratory space and also the very fact that ozone dissolves in the ALL and reacts immediately; thus, every second, more ozone reacts so that in a 6-month period the cumulative dose (likely up to 150-300 mg ozone) becomes really deleterious. Similarly several months exposure to ozone or to a prolonged oxidative stress due to a chronic inflammatory disease (atherosclerosis, diabetes, cancer) can possibly raise 4-HNE plasma levels up to 5-20 μM and, in spite of continuous detoxification, they can exert pathological effects as those observed in vitro studies performed with leukemic cells [39], lens epithelial cells [40], Jurkat T cells [41] and when testing cholesterol secoaldehyde (CSeco) in cardiomyoblasts [31]. Interestingly, tolerance to ozone or 4-HNE is far more easily achieved by small and repeated oxidative stresses than after a continuous and heavy oxidation [42,43]. On the other hand, a normal endogenous 4-HNE level (0.1- 0.7 μM) appears to act as a defensive agent against itself and other toxic compounds [13,44]. Thus, the biological behavior of HNE is an enlightening example of how the physiological serum level of a potentially toxic aldehyde produced by the normal peroxidation is proficiently used for maintaining homeostasis.

Finally, it is worthwhile to mention that the vast skin surface, possibly exposed for hours to ozone and UV radiation, can contribute to the overall toxicity: several studies performed by exposing hairless mice to ozone have shown not only depletion of the skin antioxidants but the induction of a remarkable oxidative stress [22,23]. As a consequence, humans, living in hot countries and during summer, become particularly susceptible to ozone and UV irradiation. On the contrary, a quasi-total (excluding the neck and the head) exposure of human volunteers to a very low ozone concentration in a sauna cabin for 20 min results in a very transient increase of LOPs in the peripheral circulation that exerts therapeutic effects in chronic limb ischemia's patients [45,46] interpreted as due to an induction of antioxidant enzymes and HO-1. In conclusion, although ozone is not the only culprit for adverse health effects, it significantly contributes to exacerbate respiratory illnesses and enhances mortality in about 40% of the total US population [21]. The problem is linked to the abnormal ozone concentration of troposheric ozone and to the continuously increased production of noxious compounds due to the excess burning of coal, oil and virgin forests. During this century, if human activities continue to release in the atmosphere the actual amount of global emissions, the Earth will experience a new Paleocene-Eocene Thermal Maximum or *"a fever period" *with dramatic consequences. The overall toxicity, due to the constant aggressiveness of ozone on lungs and partly on the exposed skin, associated with the relative efficiency of the detoxifying system, progressively overwhelmed by the perennial stress, favours pathological effects such as inflammation and cell degeneration particularly on lungs, liver (fibrosis), heart, kidneys and brain [47,48]. Consequently vital organs can be envisaged to be subjected to a kind of a "toxic rain" produced in the pulmonary system. Obviously, this knowledge has popularized the idea of ozone toxicity but, in the next section, it will be clarified that the generalization of this concept is incorrect.

## 2. How an appropriate ozone dose acts on human blood?

During the period 1970-2000, the ozonated autohemotherapy had been performed in an empirical fashion owing to imprecise ozone generators and the lack of knowledge of the mechanisms of action of ozone in human blood. However, by 1998, two crucial events changed the outlook: firstly, new ozone generators with precise photometers able to accurately measure (at 253.7 nm) the ozone concentration became available, and secondly, it was clarified how ozone works when mixed with blood ex vivo [49,50]. Oxygen equilibrates with the extracellular and intraerythrocytic water before becoming bound to hemoglobin until it is fully oxygenated as shown by the rapid increase of the pO_2 _from about 40 up to 400 mmHg. Ozone, ten fold more hydrosoluble than oxygen, readily dissolves in the aqueous environment of plasma and is partly (between 20 and 40%) quenched by hydrophilic antioxidants such as reduced glutathione, ascorbic and uric acids acting as sacrificial compounds, while the bulk reacts with PUFA transported by the albumin. The "therapeutic window" has been carefully determined and ranges between 10 and 80 μg/ml (0.21-1.68 μmol/ml) ozone per ml of blood. It ensures a small and precise oxidative stress able to elicit medical efficacy, but no toxicity. It must be noted that ozone acts as a pro-drug because, during this fast reactions, ozone disappears but generates two crucial messengers: the first is H_2_O_2 _and the second is a mixture of LOPs finally exemplified by 4-HNE (from omega-6 PUFA) and 4-HHE (trans-4 hydroxy-2-hexenal from omega-3 PUFA).

The behavior and pharmacodynamic of H_2_O_2 _have been ascertained: the initial formation of a gradient between plasma and intracellular water [51] allows its entrance into the erythrocytes, lymphocytes and platelets, but its concentration remains around a few micromoles because it is quickly reduced to H_2_O by free GSH, catalase and GSH-Px [52]. Its half-life is less than 60 seconds and yet its intracellular concentration is critical, because in order to activate some biochemical pathways (formation of GSSG with consequent activation of the pentose cycle in the red cell and activation of a tyrosine kinase in lymphocytes), it must reach a critical threshold that, nonetheless, is not cytotoxic. The concept of threshold is physiologically important and means that an ozone dose below 10 μg/ml of gas per ml of blood, in most cases, is biologically ineffective because the ozone dose is totally neutralized by the plasma antioxidants. In other words, the concept of a threshold helps to understand that a too low ozone dose may be ineffective (placebo effect) while a dose higher than the therapeutic one can be toxic. It is almost needless to add that saline-washed erythrocytes suspended in saline, even if exposed to very low ozone concentrations, undergo conspicuous hemolysis, an artificial result [53] that has favoured the concept of ozone toxicity. Provided that the ozone dose is within the well defined range, there is only a transitory decrease (no more than 25%) of the potent antioxidant capacity of plasma [54] fully reconstituted within 20 min owing to the efficiency of the redox system [32]. There is neither damage to erythrocytes nor to other cells: hemolysis is negligible (from 0.4 up to 1.2%), there is no leakage of K+ and methemoglobin remains normal [55]. It must be added that ozonated erythrocytes show an improved glycolysis with an increase of ATP and 2,3-DPG levels, which are able to shift the dissociation curve of HbO_2 _to the right, confirming the observation of an improved delivery of oxygen in peripheral obstructive arterial disease. Extensive data have been reported in reviews [56-58] and two books [46,50]. It is now clear that a "physiological" ozone dose (most frequently ranges between 20 and 40 μg/ml ozone per ml of blood) triggers an acute and precisely calculated oxidative stress able to activate several biological processes summarized in Table 1. What happens during the rapid infusion of the hyperoxygenated-ozonated blood into the donor ? The hyperoxygenation of blood (pO_2_, at about 400 mmHg) is irrelevant because, during the 15-min infusion period, it mixes with venous blood so that the final venous pO_2 _relative pressure is hardly modified. Oxygen-ozone behaves similarly when this gas mixture comes in contact with either a moist human skin [45] or the rabbit colon-rectal mucosa [59], ozone dissolves immediately in the water overlaying the epithelium and reacts with either sebum, or mucoproteins, faeces and any other biomolecules present in the liquid film generating hydrogen peroxide and LOPs. Only LOPs are absorbed via lymphatics and venous capillaries and reach first the liver and then enter into the general circulation where these have been measured [59].

**Table 1:** Ozone therapy can induce the following biological responses

a)	Improves blood circulation and oxygen delivery in ischemic tissue owing to NO, CO, and increase levels of intra-erythrocytic 2, 3-DPG.
b)	Enhances general metabolism by improving oxygen delivery.
c)	Upregulates cellular antioxidant enzymes and induction of HO-1 and HSP70.
d)	Induces a mild activation of the immune system and enhances the release of growth factors.
e)	Does not procure acute or late side effects.
f)	Procures a surprising wellness in most patients, probably via the stimulation of the neuroendocrine system.
g)	Activates neuroprotective systems.

References: [46,58,81,84,119]

The next important step was to evaluate the distribution, the fate and the biological significance of alkenals. After the 5 min mixing blood with the gas mixture *ex vivo, *the ozonated blood is ready to be infused back into the donor patient. Both ozone and hydrogen peroxide have been exhausted but alkenals have formed adducts with either the Cyst 34 of albumin or/and GSH and will be infused into the patient's circulation. They will interact with endothelial cells and then with billions of cells of various organs [46]. Just in case an inexpert ozone therapist uses an excessive ozone dose, he may oxidize Cys 34 to sulfenic acid: RSOH, which however can be reduced again.

The distribution of 4-HNE and similar alkenals has pharmaco-toxicological relevance: owing to the intrinsic toxicity of aldehydes, it is important to know their metabolism and fate: Alary et al. [60] have reported that about 70% of 4-HNE is excreted in urine within 2 days after its intravenous administration in rats. Siems and Grune [61], after investigating the metabolism of 4-HNE in several mammalian cells and organs, demonstrated that 4-HNE, at a concentration of 100 μM, was degraded within 3 min of incubation at 37°C, and it took only 10-30 s to restore the physiological level of about 0.2 μM. The kinetic of disappearance from mildly ozonated blood of thiobarbituric acid reactive substances (TBARS), including MDA and 4-HNE has been measured in six patients with age-related macular degeneration (ARMD), and their half-life was equivalent to 4.2 ± 1.7 min [62]. On the other hand, when the same samples were incubated in vitro (at 37°C and pH 7.3), LOPs levels hardly declined during the next 9 h, indicating their stability in an acellular medium and suggesting the relevance of cellular catabolism [49]. On the whole, it appears that mammals have developed an efficient detoxification machinery to metabolize 4-HNE and minimize its toxicity: Awasthi et al. [42] not only have indicated six enzymes: glutathione S-transferases, aldoketoreductases, aldose reductase, aldehyde dehydrogenases Cyp450 4A and β-oxidation enzymes, important in the metabolism of 4-HNE, but they and other authors [40,43,44] have emphasized that 4-HNE stress-preconditioned cells can develop a significant adaptive response by upregulating the synthesis of γ-glutamate cysteine ligase, γ-glutamyltransferase, γ-glutamyl transpeptidase, HSP-70, heme oxygenase-1 and a variety of antioxidant enzymes. There is now ample consensus on the importance of the induction of cell tolerance to submicromolar levels of 4-HNE [13,63-66]. At this point, it seems useful to point out that mammalian organisms, for controlling 4-HNE toxicity due to oxidative stress and maintaining it at physiological plasma level of 0.3-0.7 μM enact the following processes:

a) *Dilution*, a simple calculation indicates that a bolus injection of a dose of 500 μM 4-HNE in 10 ml of plasma, once diluted in a plasma-extracellular fluid volume of 12 l, irrespective of any other process, yields a concentration of as low as 0.04 μM.

b) *Detoxification, *due to the direct inactivation of 4-HNE with GSH and ascorbate or to the interaction with billions of cells endowed with detoxifying enzymes [42]

c) *Excretion, *into bile and urine after hepatic detoxification [67] and renal excretion [60], and

d) *Cell internalization*, this is a crucial and interesting point because the consequent biological effects can be either negative or positive. Several months exposure to inhaled ozone or to a prolonged oxidative stress due to a chronic disease (atherosclerosis, diabetes, inflammation) can possibly raise 4-HNE plasma levels up to 5-20 μM and, in spite of continuous detoxification, they can exert pathological effects. Interestingly, tolerance to ozone or to 4-HNE is far more easily achieved by small and repeated oxidative stresses than after a continuous and heavy oxidation [42.43]. On the other hand, a normal endogenous 4-HNE level (0.1- 0.7 μM) appears to act as a defensive agent against itself and other toxic compounds. *At this stage, alkenals acquire the great value to hypothetically trigger the molecular mechanisms leading to elicit medical efficacy without toxicity. *This aspect is discussed in the next section.

## 3. Oxidative stress and nuclear transcriptional factors in carcinogenesis

Chronic inflammation plays a critical role in neoplastic growth (transformation), as well as in many other diseases. Inflammation is induced via NFκB activation by various inflammatory factors, such as LPS and severe oxidative stress derived from drugs that induce iNOS, COX-2, and inflammatory cytokines. These factors induce the survival, growth, and proliferation of tumor cells, and result in neoplasia [68]. Therefore, inhibition of NFκB is an important target for the prevention and treatment of cancer. For example, it is known that the inhibitors of NFκB, such as vitamin E, resveratrol, curcumin, catechin, and aspirin may delay or suppress tumor growth.

### 3.1. Anti-neoplastic mechanisms of Nrf2

**Figure 1:**
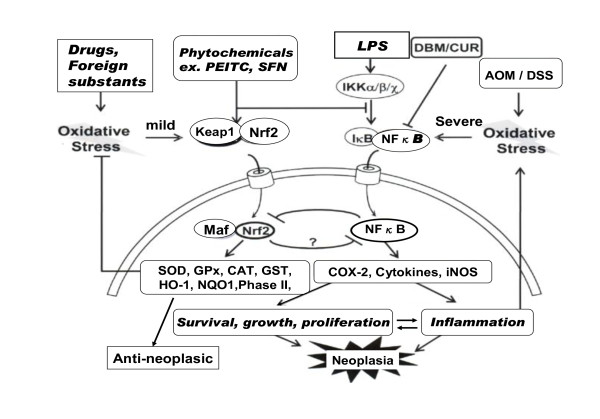


Chronic inflammation plays a critical role in neoplastic growth as well as in many other inflammatory diseases. Therefore inhibition of NFkB is an important target for the prevention and possibly treatment of cancer. Has Nrf2 a role in ozone therapy ? Li et al [69] demonstrated that Nrf2, an important cytoprotective nuclear transcription factor, suppresses NFκB-activation. As shown in Fig.1(left side), Nrf2 is usually present within the cytosol as a complex with Keap-1 protein. The question is: are alkenals able to dissociate this complex ? The Keap-1 protein has two SH-groups and the formation of adducts may cause a conformational change favoring its dissociation. Besides other pathways to be experimented and a mild oxidative stress, Nrf2 is released from this complex and is transported into nucleus. The transported Nrf2 forms a new complex with Maf protein, and induces the transcription of various antioxidant and phase II detoxification enzymes by binding to antioxidant response element (ARE) on DNA. The specific antioxidative enzymes that are activated, include SOD, catalase (CAT), GSH, GSH-reductase, GPx, GSH-S-transferase (GSTr), HO-1, NADPH quinine-oxidoreductase 1 (NQO1), heat shock protein 70 (HSP70), and phase II enzymes. These enzymes may elicit anti-neoplastic effects.

Sulforaphane (SFN), a component in broccoli is the most common phytochemical that induces the transcription of various antioxidant enzymes via the activation of Nrf2. In fact, SFN alone may also act as an oxidative stressor in cells.

### 3.2. Anti-neoplastic phytochemicals are not effective in Nrf2 knockout mice

In the previous section, the anti-neoplastic effects of phytochemicals, such as SFN, have been explained with respect to their via activation of Nrf2. It is well known that SFN possesses potent chemopreventive effects through the induction of cellular detoxifying/antioxidant enzymes via the Nrf2 [70]. If these effects are really mediated via Nrf2, then the actions of SFN should be inactived in Nrf2 knockout mice, and active in wild-type mice. Indeed, this observation was made by Xu et al [68] when they examined the chemopreventive efficacy of SFN in Nrf2 wild type (+/+) and Nrf2 knockout (-/-) mice. A topical application of 100 nmol of SFN once a day for 14 days prior to 7,12-dimethyl-benz(a)anthracene/12-O-tetradecanoyl-phorbol-13-acetate treatments decreased the incidence of skin tumors in the Nrf2 (+/+) mice compared to the vehicle-treated group. More importantly, there were no chemopreventive effects elicited by SFN pre-treatment in Nrf2 (-/-) mice. Taken together, these results demonstrate, for the first time, that Nrf2 (-/-) mice are more susceptible to skin tumorigenesis, and that the chemopreventive effects of SFN are mediated via Nrf2 activation.

### 3.3. Oxidative stress induced by moderate exercise induces Nrf2 -activation

Moderate and severe exercise results in the production of ROS in both muscle and internal organs. ROS are not only toxic, but also play an important role in cell signaling and the regulation of gene expression. Moderate exercise is known to be good for health, as opposed to severe exercise. However, the mechanisms of action of the ROS produced during exercise are not fully understood.

The production of ROS during exercise is primarily due to disorders of the mitochondrial electron transport system induced via an increase in oxygen consumption, elevations in NADPH-oxidase activity resulting from ischemia and reperfusion of internal organs, and inflammation induced via the infiltration of inflammatory cells, such as macrophages and neutrophils. ROS production levels may also depend on the intensity of exercise.

Gomez-Cabrera et al [71] reported that moderate exercise results in the activation of MAP kinases and the NFκB pathway, and consequently the expression of eNOS, iNOS, and MnSOD. Interestingly, all of these changes were abolished when ROS production was inhibited with allopurinol, an NADPH-oxidase inhibitor. These findings suggest that exercise-induced ROS production results in the expression and activation of antioxidant enzymes, such as MnSOD and GPx, primarily via the activation of the NFκB pathway. However, evidence regarding the induction of antioxidant enzymes via the NFκB pathway is still lacking. Thus, we cannot reject the possibility that Nrf2 is also activated. Thus, to hypothesize that moderate exercises induces the expression of antioxidant enzymes, and that ROS production induces the activation of NFκB and Nrf2 may be reasonable, but it remains to be clarified.

### 3.4. Biological responses induced via the activation of Nrf2/Keap1/ARE

Nrf2 is activated by mild oxidative stress, and increases the transcription of ARE [72,73]. This may be a possible mechanism behind the anti-neoplastic effects of this pathway. Other potential biological responses elicited via an activation of the Nrf2/Keap1/ARE pathway are summarized in Table 2. Items 1 to 7 were already discussed by Dinkova-Kostova [74], whereas items 8 and 9 will be discussed in the next sections.

**Table 2:** Biological responses induced via the activation of Nrf2/ARE with mild oxidative stress

1)	Increasing the levels of direct antioxidants, such as GSH, CO, and bilirubin.
2)	Stimulating GSH regeneration via glutathione and thioredoxin reductase.
3)	Increasing the levels of enzymes that detoxify oxidants and electrophils (i.e. catalase, SOD, GPx, GSTr, NADPH-quinone oxidoreductase (NQO1), HO-1, HSP70, etc).
4)	Increasing the levels of phase II enzymes.
5)	Inhibiting cytokine-mediated inflammation via the induction of leukotriene B4 reductase.
6)	Reducing iron overload, and subsequent oxidative stress induced via elevated ferritin.
7)	Recognizing, repairing, and removing damaged proteins.
8)	Protection from apoptosis induced via oxidative stress*.
9)	Increasing DNA repair activity*.

Ref from 1) to 7): Dinkova-Kostova AT [74].

References from 8) to 9) : Villeneuve NF. [78].

### 3.5. Nrf2 is associated with apoptosis and DNA repair

Numerous reports suggest that oxidative stress induces cell apoptosis, and antioxidants, such as increased levels of HO-1, protect the cell against apoptosis. HO-1 is induced by Nrf2, and prevents against apoptosis. For example, the complex work of Brunt et al [75] demonstrated that apoptosis of human vascular smooth muscle cells is induced by H_2_O_2_, and that the activation of the HO-1 promoter via Nrf2 protects these cells from the apoptotic effects of H_2_O_2_. Furthermore, apoptosis induced via either chromium (VI) [76] or LPS [77] also protected by Nrf2-dependent HO-1 induction. These findings suggest that anti-apoptotic effects are due to the activation of antioxidant protection systems induced via the activation of Nrf2.

Nrf2 has been also shown to be involved in DNA repair (#9 in Table 2). Villeneuve et al [78] found that Nrf2 is significantly associated with DNA repair. In fact, both Nrf2 and p21 were found to regulate the fine balance between life and death by controlling ROS levels. That is, the cytoprotective effects, specifically those concerning DNA repair or apoptosis, are likely dependent on the magnitude of oxidative stress. Therefore, p21-mediated activation of the Nrf2 signaling pathway may be the first defense mechanism used to reduce ROS under low stress conditions. At low levels of oxidative stress, p21 activates the Nrf2-dependent antioxidant response, which protects the cell from ROS-induced cellular damage. At moderate levels of oxidative stress, which involves DNA damage, p21 induces cell cycle arrest to allow for DNA repair. At high levels of oxidative stress, or at the point of no return, Nrf2 is destroyed, and thereby, the Nrf2-dependent pro-survival response is inhibited, and the pro-apoptotic response initiates apoptosis.

In conclusion, the Nrf2-dependent antioxidant response has been shown to protect against oxidative-stress related diseases, such as cancer [79,80] neurodegenerative diseases [81,82], cardiovascular disease [83-85], lung emphysema [86], inflammation [87,88] and aging [89]. This background will be useful for evaluating the role of Nrf2, but its actual relevance remains to be demonstrated by selected experiments connected with ozone therapy.

## 4. Biological responses and therapeutic effects induced by ozone therapy

The versatility of ozone therapy applications is impressive but there are some limitations, and therefore ozone therapy is not a panacea. From the available data, vascular and some degenerative diseases are the most suitable to be treated, while acute and chronic infectious diseases such as HIV infection and chronic hepatitis C cannot be cured with ozone therapy, because both viruses and other pathogens, either free in the plasma or/and present insides cells, cannot be inactivated by the ozone messengers and actually are well protected by the potent antioxidant capacity of blood.

### 4.1. Clinical results in peripheral obstructive arterial diseases (POAD)

Since the initial work of Rokitansky [90], several small clinical studies have been performed on POAD showing a consistent improvement of the local circulation after a cycle of 13-18 ozonated AHT [reviewed in Bocci, 46]. It is well known that even a modest obstruction of limb arteries due to atherosclerosis, diabetes, or Buerger's disease (thromboangiitis obliterans) leads to a progressive reduction of blood flow to the limb.

Tissue ischemia and any minor trauma facilitate the formation of an ulcer, which will no heal because of reduced oxygenation, lack of nutrients and growth factors indispensable for the repair process. Ozone therapy firstly lead to the activation of glycolysis with an increase in adenosine triphosphate (ATP) and 2,3-diphoshoglycerate (2,3-DPG). Consequently the sigmoidal oxygen-binding curve of Hb shifts to the right and increases the release of oxygen in the ischemic tissues. The modest but critical entrance of H_2_O_2 _in the erythrocyte is promptly reduced it to water through the action of GSH. The increased formation of oxidized GSH as GSSG alters the GSH/GSSG ratio but this change is rapidly corrected by either expelling some of the GSSG, or by reducing it via GSH-reductase at the expense of either ascorbic acid or thioredoxin. It is noteworthy that this critical recycling proceeds within 3 minutes [32]. Moreover the activation of glucose-6-phosphate dehydrogenase (G6PDH) can provide reducing power and activates glycolysis [91]. It must be noted that the metabolic profile of ozonated blood is practically over imposable with that of simply oxygenated blood [92].

According to the Fontaine-Leriche classification, patients at either stage II (intermittent claudication and transitory pain), or stage III (continuous pain, cyanosis, and possibly initial ulcers) achieve the best results. Stage IV includes incipient necrosis of toes and unbearable pain leads to surgical amputation that can be reduced or delayed with ozonated-AHT in about 50% of cases. In comparison to pentoxyfilline and prostanoids (the gold standard of orthodox treatment), ozonated AHT has proved more effective and without side effects in ischemic vascular disease. There is no substantial difference in the results either using AHT or the extravascular ozonation of blood, which is an invasive and expensive method. By using the former procedure [93.94], 28 patients, randomized to either receive their own ozonated blood or a cycle of prostacyclin infusion were evaluated. All patients continued conventional treatment with statins, anti-hypertensive and anti-platelet aggregation drugs. Ozone therapy proved more effective than prostacyclin in terms of pain reduction and improvement of the quality of life, but no significant difference was seen in vascularization of the lower limbs in either group, most likely due to the short duration of treatment (14 treatments in 7 weeks). In this complex pathology, more prolonged treatments asscociated with topical therapy with ozonated oil may led to a satisfactory healing of ulcers, because it is a mistake to stop therapy too early. Actually this therapy should be continued for life with less frequent treatments. An improved schedule on a trial in progress consists of two ozonated-AHTs (225 mL blood plus 25 mL of 3.8% sodium citrate solution), given weekly for at least 4 months. In patients at stage III and IV, the topical therapy is most important when performed with ozonated water and olive oil because it helps to accelerate healing of ulcers. How well ozonated-AHT works it appears evident by the fact that the nocturnal excruciating pain disappears after the first two to three treatments, indicating the improvement of blood flow in the ischemic tissue and the lack of ''stealing'' blood away from underperfused muscle.

An experimental confirmation of Nrf2 activity regarding the positive mechanisms inducing the antioxidative protection systems, is warranted. Furthermore, Nrf2 may be a potential mechanism involved in the upregulation of cellular antioxidant enzymes and induction of HO-1 and HSP70 (Table 1, c). It has been already shown that therapeutic ozone concentrations can induce both HO-1 and HSP70 [95], but the role of Nrf2 remains to be ascertained. It seems evident that the therapeutic activity of ozone in POAD is due to an improvement in blood flow and oxygen delivery in the ischemic areas. Moreover it was recently demonstrated that a nuclear transcription factor, hypoxia inducible factor-1α (HIF-1α), may participate in improving the outcomes of ischemic tissues [96-98]. HIF-1α, an ubiquitously expressed molecule, regulates a number of genes (i.e. hypoxia response elements, HREs) that elicit compensatory responses during states of hypoxia, metabolic compromise, and oxidative stress. Activation of HIF-1α results in increases of vascular endothelial growth factor (VEGF), erythropoietin (EPO), and glycolytic enzymes, all of which enhance cell proliferation and survival. Furthermore, an increase in the biosynthesis of VEGF can stimulate neoangiogenesis, and consequentially improve blood flow, and increase the biosynthesis of erythropoietin, which augments oxygen delivery. An increase in glycolytic enzymes enhances glucose metabolism, and consequently increases the biosynthesis of NO and CO, which also stimulate blood flow and oxygen delivery into hypoxic tissues. Thus, these biological responses may induce the excellent therapeutic effects of ozone-induced mild oxidative stress in POAD. Additionally, points a) and b) in Table 2 may be explained by the role of HIF-1α. In the normoxic state, HIF-1α is inactivated by proline hydroxylases (PHDs) and VHL within the cytosol. On the other hand, in hypoxia, PDHs and VHL are inactivated by mild oxidative stress, and HIF-1α is released from PHDs and VHL, transferred into nuclei, where HIF-1α forms a heterodimer with HIF-1β, and results in the transcription of hypoxia response elements (HREs) on DNA. It was reported that about 2000 genes in HREs are regulated by HIF-1α [99] including genes involved in the regulation of apoptosis, pH regulation, and proteolysis, as well as angiogenesis, erythropoiesis and glucose metabolism. Another remark regards the postulation that 4-HNE reaching the bone marrow environment may favor the release of staminal cells, which may be able to switch on angiogenesis in the ischemic tissues. It is hoped to clarify if any of these hypothesis are valid in the near future.

Millions of people suffer from chronic limb, brain, and heart ischemia, which represent the major cause of death worldwide. This has a huge socio-economic impact, particularly in the developing world. If only orthodox medicine will accept ozonated AHT as an adjunct to standard medication, a great leap forward will be noted. It is most unfortunate that owing to the lack of sponsors and funds it has not yet been possible to carefully evaluate controlled clinical results in either stroke or chronic heart failure patients

### 4.2. The relevance of ozone therapy in Age-Related Macular Degeneration (ARMD)

In the UK alone, some 250,000 patients affected by the ''dry'' (atrophic) form of ARMD are suitable for treatment with ozonated AHT, but all over the world there are more than 30 million people searching for a therapy. Nonetheless, ophthalmologists can only prescribe antioxidants and zinc, which are minimally effective. Since 1995, almost 1,000 patient with the dry form of ARMD have been treated with ozonated AHT at the Siena (Italy) polyclinic and three-quarters have shown an improvement of one to two lines on the visual acuity chart (reviewed in 46). Moreover it is relevant that the disease does not progress during ozone therapy. Usually 15-18 treatments, at an initial ozone concentration of 20 mg/ml of gas per ml blood, slowly upgraded to 60 mg/ml (twice weekly), followed by two monthly session as a maintenance therapy, allows to maintain the improvement. Although uncontrolled, this study emphasizes that ozone therapy is the only treatment able to dramatically improve the patient's quality of life. In this disease there is progressive degeneration and death of the fovea centralis photoreceptors and of the pigmented retinal epithelium (PRE) as a consequence of several factors, one of which is chronic hypoxia. Although O_3_-AHT induces a pleiotropic response, the main advantage is due to an increased delivery of oxygen to the retina, which is the bodily tissue with the highest oxygen consumption. It is worth noting that AHT is useless, even harmful, in the exudative form of ARMD and in multigenic and progressive disorders (e.g., retinitis pigmentosa and recessive Stargardt's disease). The exudative form, characterized by an aberrant choroidal vascular growth and a vascular hyper-permeability beneath the retina and the PRE, is caused by worsened ischemia, which stimulates the release of the vascular endothelial growth factor [99]. In the exudative form it seems that the intravitreous administration of anti-VEGF antibody may temporarily slow down angiogenesis with a slight functional improvement. It must be emphasized that O_3_-AHT (in the dry form) not only improves visual activity but at least, in part, helps the patient to re-acquire the capacity of autonomous life. Finally it is interesting to inform that only during the last month a preliminary trial evaluating whether the intravitreal injection of staminal PRE in blind patients with Stargardt's disease will be able to restore vision. Although patients will receive an immunosuppressive therapy, there are other risks but after ten years of preliminary studies, the trial needed to be performed.

### 4.3. The effects of ozone therapy in diabetes

Diabetic nephropathy induced via diabetes mellitus is linked with oxidative stress. The potential therapeutic effects of ozone therapy were studied in streptozotocin (STZ)-induced diabetic rats [100]. The induction of diabetes mellitus significantly elevated blood pressure, HbA1c, BUN, creatinine, and renal tissue levels of MDA, whereas SOD, CAT and GPx activities were significantly reduced. Treatment with either insulin or ozone therapy significantly reversed the effects of diabetes mellitus. Furthermore, a combination of both insulin and ozone therapy even further reversed the effects of diabetes mellitus in comparison to monotherapy. Thus, ozone administration in diabetes mellitus rats reduced levels of oxidative stress markers and improved renal antioxidant enzyme activities, especially when rats were treated with a combination of ozone and insulin. This initial result suggested that ozone therapy may be useful for treating diabetic patients, specifically by mediating antioxidative responses. Clinical evidence that ozone therapy is useful has been anecdotically reported several times by private ozone therapists but a valid, controlled clinical trial has yet to be performed. Chronic limb ischemia is often accompanied by type 2 diabetes and these patients have been advantageously treated with ozonated AHT [46]. The need of reducing the insulin dose, suggesting either an improved insulin secretion or/and an increased receptor sensitivity has become a common observation.

As ozone is used in all Cuban hospitals, Cuban ozone therapists have performed a randomized controlled clinical trial in diabetic patients with peripheral arterial diseases and diabetic foot [101]. One group (n = 51) was treated with ozone administered via rectal insufflation of 200 ml of gas with an ozone dose of 10 mg together with topical ozone both as gas and ozonated oil. The control group (n = 49) was treated with systemic and topical antibiotics. The efficacy of the treatments was evaluated by comparing the glucose level, the area of the ulcers and several biochemical markers after only 20 days of treatment. A drawback was the lack of a follow-up after the 20 days therapy. The experimental group, after receiving a total dose of 200 mg ozone showed an improved glycemic control, a reduced oxidative stress, an increase of SOD, a significant improvement of the lesions without any side effects and with a fewer amputations than the control group. Human diabetic foot is a chronic disease and these results are even more surprising not only because they were achieved only after 20 days therapy, but also because among the five routes of ozone administration, [reviewed in 102], i.e.: (1) the major and minor ozonated autohemotherapy; (2) the extracorporeal circulation of blood against oxygen-ozone; (3) the quasi-total body exposure to oxygen-ozone; (4) the intracavitary, subcutaneous, intramuscular administration, and (5) the ozone insufflation via rectum is the most unreliable administration route because we never know the percentage of the really effective ozone dose insufflated into the rectum. Moreover in Europe at least 40% of patients: (i) refuse the administration of ozone via rectum; (ii) part of the ozone dose may be unwillingly eliminated immediately after the gas introduction; (iii) the fecal content and the abundant mucoproteins certainly neutralize part of the ozone dose, and (iv) in all cases ozone, unlike oxygen, is not absorbed by the rectal mucosa. In previous rabbit experiments [59], it was clarified that only peroxidation products could be detected in portal blood. This is because ozone immediately reacts with a variety of biomolecules present in the luminal content and only some of the generated compounds are absorbed and may exert therapeutic activity. The concentration of ozone (50 mg/ml) used in the Cuban patients is too high and unwise because it can cause painful intestinal cramps. Moreover it must keep in mind that Eliakim et al. [103], after repeated enema in rats with ozonated water, have reported the appearance of a microscopic colitis. As a consequence, even though results are spectacular, they need to be confirmed, not only by repeating the trial, but preferably by using the reliable strategy of ozonated autohemotherapy [104]. On this basis it appears urgent to organize an appropriate clinical trial in order to evaluate whether an initial cycle including at least 32 treatments during four months (twice weekly) can modify critical parameters including glycemic and C-reactive peptide levels, nonenzymatic glycosilation, aldose reductase activity, advaced glycated endproducts (AGEs) and the antioxidant-pro-oxidant balance. Although more expensive, the precise stoichiometry of the ozonated AHT method can give far more precise information than the rectal insufflation. The adopted strategy 'start low, go slow' appears the most idoneous for inducing ozone tolerance and the rebalance of the redox system [46]. Bearing in mind that O_2_-O_3 _therapy may modify glycemic levels, a strict control of the insulin level is imperative.

A debate on a scientifically proved application of a complementary approach based on the knowledge that O_3_-AHT can abate the chronic oxidative stress, delay serious complications and improve the quality of life of diabetic patients has been proposed [104]) and is urgent.

### 4.4. Possible mechanisms for activating the immune system by ozone therapy

T-cells serve a very important role in protecting our body against foreign components: when the T-cell antigen receptor (TCR) recognizes any foreign antigens, the ZAP-70 molecule undergoes a tyrosine-phosphorylation, and then activates phospholipase C γ1 (PLCγ1) [105]. Activated PLCγ1 hydrolyze phosphatidylinositol-4,5-bisphosphate (PIP2), a membrane lipid, to produce second messengers, inositol triphosphate (IP3) and diacylglycerol (DG). IP3 binds to the inositol triphosphate receptor (IP3R) on the endoplasmic reticulum (ER) membrane, resulting in a release of Ca^+2 ^from the ER into the cytosol. Increases in cytosolic Ca^+2 ^levels activate calcineurin (CN), a phosphatase dependent on Ca^+2^/calmodulin, which dephosphorylates nuclear factor activated T-cells (NFAT) and transports it into the nucleus. NFAT then induces the transcription of cytokines, such as IL-2, TNFα, IL-6 and IFNγ, and immune response elements on DNA, which are, then, translated into their proteins [106]. The produced cytokines and transcription products of immune response elements support the immune functions of our body. NFAT is also expressed in other cells and tissues, and can itself combine with DNA elements. However, the combination of NFAT with DNA is primarily induced via the coexistence of activated protein-1 (AP-1), a nuclear transcription factor. AP-1 is activated through the Ras-MAPKs (mitogen activated protein kinases) pathway, which is also mediated by DG.

It was previously reported that immunosuppressive drugs, such as cyclosporine A and KF506 (tacrolimus), can inhibit the de-phosphorylation of NFAT induced by CN, and thereby the transportation of NFAT into the nucleus [107]. Ca^2+^-antagonists, such as anti-hypertensive drugs, inhibit Ca^+2 ^influx into the cytosol and the constriction of vascular smooth muscle cells. However, several epidemiological studies suggest that prolonged use of Ca^2+^-antagonist may result in tumors or the development of chronic diseases. Ca^2+^-antagonist inhibits Ca^+2 ^influx into cytosol of T-cells, and the activity of CN. Thus, they also may induce the de-phosphorylation NFAT, and its transportation into the nucleus. Consequently, the biosynthesis of cytokines would also be inhibited. This may explain the why long-term use of Ca^2+^-antagonist may result in tumors or the development of chronic diseases. Furthermore, it is well known that these nuclear transcription factors are also induced by mild oxidative stress. For example, Maziere et al [108] found that high levels of UVA radiation inhibit the CN-NFAT system, and low levels of UVA activate the CN-NFAT system. Therefore, mild oxidative stress induced by ozone therapy may also activate NFAT and AP-1 to then activate immune functions. This may explain of point d) in Table 2. However it remains to be seen whether a mild ozone therapy does really activate the CN-NFAT system

Therefore, beside this possible pathway, it must be kept in mind that several studies [109-111] have been carried out by using human normal blood treated with ozone concentrations within the "therapeutic window". With these parameters ozone acts on human blood and yields a hormetic dose-response relationship without any toxicity [112]. After incubation of up to 8-9 hours, the plasma was isolated and several cytokines synthesized and released by immune cells such as IFNγ, TNFα, IL-2, IL-6 and IL-8 have been measured and their small but consistent amounts were ozone-concentration dependent. During each ozone therapy session, this induced bland immuno-stimulation regards only about 4% of the lymphocytes and monocytes present in the blood exposed to ozone ex vivo [46]. It was postulated that the small percentage of immunocytes activated *ex vivo *by H_2_O_2_, via NFkB may transfer the activation in vivo after the infusion of ozonated blood into the donor patient. Indeed, after blood infusion into the donor, the activated lymphocytes, by releasing cytokines, can activate other cells in vivo [46]. If this is true, repeated therapeutic sessions may indeed reverse a condition of immune-depression. Actually these observations have suggested to use ozone therapy in both acute and chronic bacterial and viral infections keeping in mind that all pathogens, either free in the plasma or located intracellularly, are paradoxically well protected by the potent antioxidant capacity of blood and cells [113]. This is so, because with the ozone concentration established by the therapeutic index, neither ozone or its messengers can deliver a bactericidal or virucidal effect. Indeed, contrary to what is commonly believed, ozone can act only as a supportive therapy in infectious diseases and we know already that both HIV [114] and chronic hepatitis C infections cannot be proficiently treated only with ozone therapy unless we simultaneously combined with the appropriate dosages of antiviral drugs.

Moreover we are totally at a loss to discover if ozone therapy can be useful in autoimmune diseases, such as rheumatoid arthritis, multiple sclerosis and psoriasis. Once again private ozone therapists have treated these diseases claiming positive results, which have been never reported in medical journals. The key problem is to discover the ideal ozone concentration for quenching the activity of cytotoxic T cells and to enhance the number and activity of CD4+ T-Regulatory cells [46].

### 4.5. May ozone kill cancer cells and have therapeutic effects?

An early paper by Sweet et al [115] elicited great enthusiasm having shown that ozone selectively inhibits the growth of a variety of human cancer cells *in vitro. *Unfortunately this approach does not reflect the situation in the patient because ozone as such is never able to reach any cancer cells *in vivo. *Another hypothetical possibility had been to restore normoxia and apoptosis of tumor cells after the infusion of ozonated blood [91], but clinical experience in pre-terminal patient with hepatic or/and pulmonary metastasis has shown the irreversibility of the cancer. An experimental attempt [116] to insufflate an ozone and oxygen mixture into the peritoneum at an advanced stage of cancer led to a higher survival rate of treated rabbits (i.e. 7/14) versus sham-treated rabbits (i.e. 1/7). In a letter, Bocci [117] have suggested that these anti-tumor effects may be induced via an ozone/oxygen-mediated activation of the body's immuno-surveillance, which is a real possibility in a virgin animal. Indeed these results are evident only in experimental oncology, particularly in mice and not in humans where, once the cancer is discovered, the immune system is already suppressed. To be objective, there are a number of rather old and uncontrolled clinical studies demonstrating that ozone therapy extends the life expectancy of patients with cancer. This is partly true in the sense that ozonated autohemotherapy improves the quality of life as often observed [46], but is unable to block the tumour progression. Chronic inflammation plays a critical role in neoplastic growth (transformation), as well as in many other diseases. NFκB is activated by various inflammatory factors, such as LPS and severe oxidative stress derived from drugs that induce iNOS, COX-2, and inflammatory cytokines. These factors induce the survival, growth, and proliferation of tumor cells, and result in neoplasia [118]. Therefore, inhibition of NFκB is an important target for the prevention and treatment of cancer. For example, it is known that the inhibitors of NFκB, such as vitamin E, resveratrol, curcumin, catechin, and aspirin may have some effects in slowing tumor growth.

### 4.6. A possible mechanism by which ozone therapy improves neurodegenerative diseases

**Figure 2:**
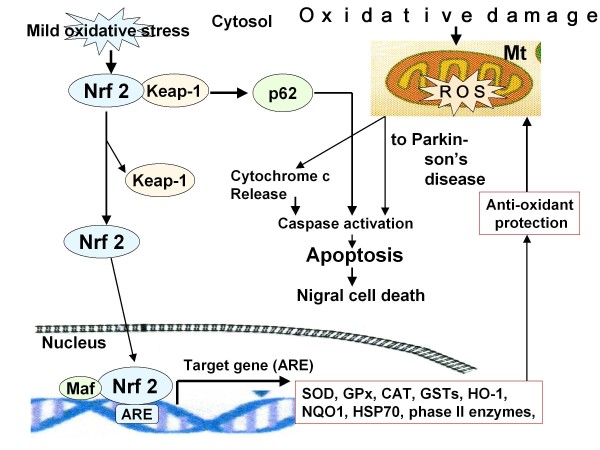


The pathogenesis of neurodegenerative diseases is significantly associated with oxidative stress. Increased oxidative stress is also associated with neuronal cell death during the pathogenesis of multiple chronic neurodegenerative diseases, including Alzheimer's disease (AD), Parkinson's disease (PD), Huntington's disease (HD), and amyotrophic lateral sclerosis (ALS). It was found that the Nrf2/ARE pathway protects against neurodegeneration [81,119] as mentioned previously. Figure 2 demonstrates that PD may result from apoptosis of neuronal cells and death of Nigral cells induced via ROS. Increases in ROS result in mitochondrial dysfunction and neuro-inflammation, a common denominator of PD. On the other hand, Nrf2 acts as an emerging target to counteract mitochondrial dysfunction and inflammation. Nrf2 activates the antioxidant response element (ARE) pathway, including a battery of cytoprotective genes, such as antioxidants and anti-inflammatory genes, and several transcription factors involved in mitochondrial biogenesis [119].

Jazwa et al [82] also found that Nrf2 and HIF-1α play a crucial role in neuroprotection and suppression of neuroinflammation in neurodegenerative diseases, especially through the activation of HO-1 via the Nrf2/ARE pathway (for HO-1 see also Idriss et al. [120]). Furthermore, drugs activating Nrf2 may also be good candidates for inducing HO-1, in concert with other antioxidant and detoxification enzymes. In fact, it was previously reported that there are many phytochemicals that are effective in protecting against neurodegenerative diseases [121-124]. These findings may explain the rationale (point g) in Table 1. Thus, further investigation into the effects of ozone therapy in activating the Nrf2/ARE pathway is warranted. Indeed several anecdotal observations have been done by private ozone therapists performing ozonated AHT in patients with initial Alzheimer's disease. It is unfortunate that these promising observation have been never published and therefore the precise parameters remain unknown. Once again orthodox medicine remains skeptical about the value of ozone therapy and this make progress very difficult.

### 4.7. The topical use of ozone therapy in skin and mucosal infections and lesions

Regarding both cutaneous and mucosal infections and lesions, both ozonated water and different gradation of standardized ozonated vegetable oils will be used twice daily until complete healing. Both ozonated water and oils have been already proved to be excellent disinfectants and healing stimulators, more effective than topical antibiotics, growth factors, only oxygenation, maggot and negative pressure wound therapies. In conclusion, this paper aims to emphasize the possibility of a new approach for improving the gloomy prognosis of the diabetic foot, ischemic ulcers and necrosis, decubitus, abscesses, anal fissures, fistulae, aphthae, inveterate osteomyelitis stomatitis, vulvovaginitis and onycomycosis. There are experimental studies [125,126] clearly showing the advantages of using ozonated oils and a great number of pertinent patents have been reviewed [127,128]. Any wound or lesions must firstly be cleaned possibly with ozonated water or diluted H_2_O_2 _solution, because the removal of purulent material or fibrin or necrotic cells markedly reduces the effect of ozonated oil because of the residual presence of biological substances [113]. To date, in western countries there is not yet the mental attitude to profitably use ozonated oil, but once a patient has tried that, he will continue to use it.

### 4.8. The problem of backache and orthopedic diseases has been successfully solved with ozone

In reality the use of ozone in hernial disk is a real breakthrough and, while the use of ozonated AHT is still regarded with suspicion, the application of ozone in low back pain is practically accepted world-wide. During lifetime low back pain is a common disturbing symptoms that can affect up to 80% of the population at least once. Under radioscopic control, 3 to 7 ml of the gas mixture (usually ozone 3% and oxygen 97%), with an ozone concentration of 30-35 μg/ml are directly administered into the centre of the intersomatic space corresponding to the disc herniation [129]. An expert neurosurgeon can do it in about ten minutes and, after a suitable rest, the patient frequently gets up amazed by the disappearance of pain.

What are the mechanisms of action ? They are quite different from those of ozone in blood. Ozone readily dissolves in the water of the *nucleus pulposus *and reacts with macromolecular glycoproteins composed of carbohydrates and polypeptide chains, namely proteoglycans and collagen types II and IV. Formation of ROS is likely followed by generation of hydroxyl radicals according to the Fenton's reaction:

$$\text{PUFA}\;+\;{\text{O}}_{\text{3}}\;+\;{\text{H}}_{\text{2}}\text{O}----------\to \text{aldehyde 1}\;+\;\text{aldehyde 2}\;+\;{\text{H}}_{\text{2}}{\text{O}}_{\text{2}}$$

because traces of catalytic iron ions can easily be released from the needle during the injection. By using the Electron Paramagnetic Resonance (EPR) spin trapping technique, the transitory formation of hydroxyl radical, which has a half life of 1 × 10 ^-9 ^sec at 37°C, has been demonstrated [130]. The hydroxyl radical is the most reactive radical attacking and breaking down any biomolecule within its reach: the rapid reabsorption of hydrolytic products and free water lead to a progressive shrinkage of the nucleus and frequently to a progressive disappearance of the herniated material. The inflammatory process induced by the hernia disappears thereafter.

An even simpler and popular approach for treating the low back pain is the administration of gas into the paravertebral muscles corresponding either to trigger points or to the metamers of the herniated disk. It is an easy approach consisting in one, or up to four injections of 5-10 ml of the gas mixture per site performed very slowly. The ozone concentration must not exceed 20-25 μg/ml, because it is painful. While the first is a direct procedure, this one is an indirect one also named as "a chemical acupuncture" by Bocci [50]. The mechanisms of action of the indirect methods have been amply discussed [131] and the therapeutic benefit is almost 80% for the direct procedure and about 73% for the chemical acupuncture. So far both methodologies have been applied from Canada to China, India and Europe and almost a million people have benefited from it.

### 4.9. The use of ozone in Dentistry

This is another surprising use of ozone mostly used in children for treating primary carious lesions using a special device isolating the tooth which is treated with ozone-oxygen for about 60 sec. This procedure sterilizes the dental lesions and allows the re-mineralization of the tooth. The procedure was invented and propagated by prof. Edward Lynch [132] and it was defined as "The revolution in Dentistry"

## 5. Quality of Life (QOL) following ozone therapy

Ozone therapy is proposed to procure a surprising wellness in most patients, in part, via the stimulation of the neuroendocrine system. The mechanisms by which ozone therapy stimulates the neuroendocrine system are not yet clear. However, it was recently reported [133] that the vulnerability of neuroendocrine cells induced via mitochondrial and oxidative stress is associated with the inhibition of the Nrf2/ARE/Keap1 antioxidant pathway, and decreased expression of antioxidant and phase I/II conjugation enzymes, most of which are Nrf2 transcriptional targets neuroendocrine system was also found to be associated with the Nrf2 pathway. It is unfortunate that so far it has not been possible to comparatively evaluate the intensity and shape of the circadian rhythm of ACTH, cortisol, growth hormone and dehydro-epi-androsterone (DHEA), in normal volunteers before and after ozonated AHT. Moreover it has been postulated a release of endorphins and possibly an increased release of serotonin for explaining the feeling of euphoria and well-being post ozone therapy

Obviously a medical evaluation on the QOL on ozone therapy is necessary. To date, Di Paolo et al., [94] only group to report on the QOL after extravascular blood ozonation and a prostaglandin analogue in POAD patients, that was in accordance with the International Quality of Life Assessment (IQOLA). Patients were scored from 0 to 4 on claudication, pain, pruritus, heavy of legs, general well-being, insomnia, appetite, weakness, sight, healing, digestion and joint pain. EBOO therapy demonstrated an excellent QOL. Thus, such QOL evaluations are important, as they also may clarify if ozone therapy is effective. Regarding the ozonated AHT, besides one venipuncture, the QOL evaluation is even better with minimal, if any, side effects [46,134,135].

## 6. Concluding remarks

During the last four decades million of O_3_-AHT have been performed in Europe without any problem and the recorded four deaths [135] were due to direct intravenous administration of O2-O3, a practice prohibited since 1984. Ozone, generated extempore from medical oxygen, must be used immediately and represents about 3% of the gas mixture. Erythrocytes, after ozonization, maintain their usual life-span in the circulation [46]. The method using neutral, sterile glass bottles under vacuum is absolutely toxic-free, the cost of the disposable set for each treatment is fifteen euros and a trained nurse can easily perform the whole procedure in about 35 min [56]. The only inconvenience is the venipuncture to which patients are nonetheless compliant. It must be emphasized that the treatment is not only perfectly tolerated but most age-related macular degeneration (ARMD) and vasculopathic patients have reported a feeling of wellness and euphoria throughout the cycle. This fact explains why the compliance of the patients remains excellent throughout the years. Almost needless to say that if ozone therapy improves the patologic condition, it must be continued by adopting the maintenance therapy (two-three monthly sessions) for an undefined period.

The application of ozone therapy in all public hospitals is still being delayed by prejudice, no fundings, commercial competition by big Pharma and above all by the incomprehensible disinterest, if not plain obstruction, by Health Authorities such as the FDA in USA and the European Medical Association. On the contrary it is commonly used in Russia, Ukraine and China where most of the patients are treated with ozone therapy. Ozone is inexpensive and no patentable and it is regretful that local Health Authorities, continuously concerned by the increased medical costs, do not want to take advantage of this inexpensive procedure. Unlike other complementary approaches, all biochemical, physiological and pharmacological mechanisms elicited by ozone are in the realm of orthodox medicine. There is already good evidence that ozone therapy is more useful in chronic limb ischemia than the golden standard (prostacyclin analogues) and patients with skin lesions are very grateful for being able to use ozonated oil. A scheme of bland ozone therapy associated to a correct life-style has been proposed for delaying aging [46,136].

As it has happened for other innovative medical approaches, thanks for the efforts of many expert physicians, ozone therapy will be eventually accepted by orthodox medicine. But this will happen only when, by performing randomized and well controlled clinical trials in suitable diseases, its validity and atoxicity will unequivocally be demonstrated.

## Competing interests

The authors declare that they have no competing interests. No funds have been available to M Sagai and V Bocci for this study.

## Authors' contributions

MS described firstly the mechanism of action of NFκB, Nrf2, HIF1α, NFAT, AP-1 in normal and pathologic conditions. And then VB described the origin, distribution and fate of ozone and clinical data of the ozone therapy. Both authors gathered references, refined the search for new information and approved the final manuscript.
